# Effect of nano clay, nano-graphene oxide and carbon nanotubes on the mechanical and tribological properties of crosslinked epoxy nanocomposite

**DOI:** 10.1371/journal.pone.0259401

**Published:** 2021-11-05

**Authors:** Sedigheh Ranjkesh Adarmanabadi, Mohammad Jafari, Seyyed Mahdi Hosseini Farrash, Mehdi Heidari

**Affiliations:** Faculty of Mechanical and Mechatronics Engineering, Shahrood University of Technology, Shahrood, Iran; University of Akron, UNITED STATES

## Abstract

The objective of this study is to investigate the effects of different nanoparticles as reinforcement in a polymeric matrix on the mechanical and tribological properties of the composite. The different particles including nanoclay (NC), nano-graphene oxide (NGO), and carbon nanotubes (CNT) with various weight percentages were incorporated into the epoxy matrix. Young’s modulus, ultimate tensile strength, strain at the fracture point, and the fracture toughness of nanocomposite samples were investigated. Besides, the tribological performance of these fabricated nanocomposites was evaluated and discussed. The results show that a significant change in the mechanical properties of the nanocomposites compared to the epoxy matrix. Also, the results reveal that the combination of NC with NGO improves the mechanical properties of graphene nanocomposites. It is found that adding NC to the NG/epoxy composite, may increase the fracture toughness up to 2 times as well as improve the ultimate tensile strength and strain at the fracture point. However, there was no significant change in Young’s modulus.

## 1. Introduction

Due to the prominent combination of low shrinkage, dimensional stability, adhesion, dielectric properties, and processing versatility, epoxies are the most common thermosetting polymer for high-performance composites and adhesives [1–3]. Furthermore, epoxy has good moisture resistance and is the best material for heavy-duty coatings application due to its superior toughness and hardness values [4,5] Nevertheless, its rigidity, brittleness, and poor resistance to crack propagation limit its applications [6]. Many attempts have been made to improve their physical properties by a variety of methods, including adding nanoparticles to polymer composite [7]. Graphene which is in the form of a hexagonal lattice of carbon atoms has outstanding properties such as high mechanical properties (e.g. Young’s modulus is 1 TPa) and superior thermal and electrical conductivities [8]. Moreover, nanoclay (NC), carbon nanotubes (CNT), and graphene have extraordinary electronic and thermal properties [9–11]. Various studies have been conducted on the effect of adding such a material on the behavior of thermostats and thermoplastic polymers [12–19].

Researchers have considered the effects of NGO on epoxy. Zhao et al. [20] developed a facile in situ polymerization method for the preparation of polyamide 6 chains grafted NGO. They found that there is a 52.6% increase in the fracture toughness of reinforced epoxy composites compared with pure epoxy. Minh et al. [21] fabricated a hybrid nanocomposite of epoxy/polyester resin and graphene nanoplatelets by a new technique. Their results showed an increase of 86.6% in the tensile strength of the new nanocomposite material by adding as low as 0.2 wt% of graphene nanoplatelets. Using a facile, scalable, and commercially viable method, Olowojoba et al. [22] prepared the reduced NGO/epoxy composites to study its mechanical and thermal properties. They showed that by adding 2.0 wt% reduced NGO, thermal conductivity would increase nearly 36%, and tensile and storage moduli improve more than 13%. Nevertheless, the tensile strengths of the composites tend to decrease with increasing GO content.

To reinforce epoxy some studies investigate the role of NC on epoxy. Chan et al. [23] prepared NC/epoxy composites to examine the mechanical properties of the samples with different contents of NC. The results revealed that compared to pure epoxy, the tensile strength and Young’s modulus of proposed composite with 5 wt% of NC increased up to 34% and 25%, respectively. Wang et al. [24] considered the effect of ultrasonic stirring time on the thermal and mechanical properties of NC/epoxy composites. It was found that as the ultrasonic stirring time rises, the maximum thermal decomposition temperatures increase, but the glass transition temperature and the storage moduli of the composites decrease. Ha et al. [25] considered the influences of silane-treated clay behavior on the fracture treatment of clay/epoxy nanocomposites by comparing the fracture parameters of silane-treated specimens with those of untreated specimens at different temperatures. Kim et al. [26] discussed the waterproof characteristics of NC/epoxy nanocomposite in adhesively bonded joints. According to their results, the tensile load capacity of the NC-filled adhesively bonded joint increased and the NC reduced water absorption into the epoxy adhesive as well as into the interface between the epoxy adhesive and the steel adherent.

Several experimental researches are also directed to find the better compatibility between CNTs and polymer matrices to make a better composite. Jagtap and Ratna [27] Fabricated an epoxy/multiwall carbon nanotubes to investigate its properties of related composites. The dispersion of multiwall carbon nanotube (MWCNT) in the epoxy matrix is significantly improved due to the use of sodium salt of 6-aminohexanoic acid (SAHA) as a modifier. The addition of optimum concentration of modified MWCNT (1 wt%) resulted in around 90 and 264% improvement in the tensile strength and tensile modulus, respectively. Subhani et al. [28] applied CNTs and nanodiamonds to reinforce epoxy for the first time. Their nanocomposites with 0.2wt.% MWCNTs and 0.2wt.% nanodiamonds showed 50% increase in hardness while modulus and tensile strength enhanced to 84% and 70%, respectively. Banisaeid [29] studied the energy absorption capacity of composites reinforced with MWCNTs under ballistic impact. It was found that reinforced composite samples containing CNTs with the carboxylic functional group, absorb 57.63% of the projectile energy and represent a better performance than one without nanotubes. The results of this study indicated that, the uniform dispersion of nanotubes in the polymer matrix can significantly affect the mechanical properties, especially tensile strength.

On the other hand, many researchers used different approaches of assembling two or three different nano fillers to improve some of the poor properties presented by single filler/epoxy. Ruiz-Hitzky et al. [30] developed a mixture of clay/graphene to study its electrical and mechanical properties. They showed that in the absence of clay a rapid decantation of Graphene Nanoplatelets (GNP) in water is observed, in the presence of sepiolite the resulting dispersions remain stable during months without syneresis effects. These dispersions lead to self-supported films of clay–GNP composites provided with reasonable electrical conductivity and mechanical properties.

Jen et al. [31] prepared epoxy nanocomposites with various MWCNT/ GNP filler ratios to study the synergistic effect of the hybrid nano-fillers on the monotonic and cyclic mechanical properties of the nanocomposites. Their experimental results indicated that the composites with a MWCNT:GNP ratio of 1:9 have the higher monotonic and fatigue properties than those with other filler ratios. Adding appropriate amount of CNTs can prevent the agglomeration of GNPs. The flexible CNTs bridge adjacent GNPs to constitute a favorable network for load transfer. Chen et al. [32] prepared a new ternary hybrid of CNT/NGO/MoS_2_ through the hydrothermal method, and its microstructure, phase composition. Results revealed EP-CNTs/GO/MoS_2_ possessed the lowest friction coefficient and wear rate than EP and its other composite coatings reinforced by single filler or binary hybrids.

Shubham et al. [33] concluded that by adding different nano particles as reinforcement in polymer composites, mechanical, physical, thermal, and other properties can be improved. They also suggested the various methods to form reinforced polymer matrix hybrid composites like hand lay-up, compression molding, spray lay-up.

As mentioned above many studies provide samples in which the mechanical properties of materials are significantly improved by adding small amounts of nano-particles. In many cases, the addition of such nano-particles is performed on polymer-matrix composites, with reported improvements in mechanical, optical, thermal or electrical properties. However, some research has criticized these results. Cicero and his colleagues [34] introduced the small amounts of Graphene Oxide (up to 1%) in PA6 with the aim of studying their effect on the fracture properties of the resulting composites. For the particular conditions analyzed here, no improvements in the fracture behaviour (in both cracked and notched conditions) have been observed (a similar conclusion may be obtained for the tensile behavior). This article was a driving force for this study to consider again the effects of this nanoparticle experimentally.

In the present study, the effects of the addition of different contents of NC, NGO, and CNT on the mechanical and tribological properties of the polymer matrix nanocomposites were investigated. Mechanical stirring with ultrasonication was utilized to achieve uniform dispersion of nanoparticles into the epoxy matrix. Young’s modulus, ultimate tensile strength, strain at the breakpoint, and fracture toughness of the nanocomposite specimens were extracted from stress-strain graphs. Furthermore, the fracture and wear mechanisms were determined by studying the surface morphology.

## 2. Experimental procedure

### 2.1 Materials

The resin used in this study was KER 828 composed of bisphenol A and epichlorohydrin supplied by Kumho P&B Chemicals, Inc. A 10% wt triethylenetetramine hardener was used as a curing agent. The dynamic viscosity of KER 828 was ranged from 12 to 14 Pa.s at 25°C with density of 1160 kg/m^3^. Research grade NGO nanoplatelets powder (purity: +99.5%, thickness 2–18 nm with less than 32 layers) was provided from US Research Nanomaterials. NC montmorillonite (particle size: 1–2 nm, density: 0.5–0.7 gr/cm^3^) was purchased from Sigma–Aldrich Chemical Company and MWCNT-COOH functionalized (purity: 97%, Content of–COOH: 1.23%, outside diameter: 20–30 nm, inside diameter: 5–10 nm, length: 10–30 um, density: ~2.1 gr/cm^3^) was used.

The weight percentages of epoxy resin, hardener, and nanostructured fillers in the fabricated nanocomposite samples were 89.50, 8.95, and 1.50% respectively. The combinations of nanostructured fillers applied in the fabrication of the samples are listed in Table 1.

**Table 1:** The weight percentage of different nanostructured fillers used in samples.

Specimens	Epoxy	NGO	NC	CNT
Pure Epoxy	100	---	---	---
NGO/Epoxy	98.50	1.50	---	---
NC/Epoxy	98.50	---	1.50	---
CNT/Epoxy	98.50	---	---	1.50
NGO/CNT/Epoxy	98.50	0.75	---	0.75
NC/CNT/Epoxy	98.50	---	0.75	0.75
NGO/NC/Epoxy	98.50	0.75	0.75	---
NGO/NC/CNT/Epoxy	98.50	0.50	0.50	0.50

### 2.2 Fabrication process

The process of preparing epoxy composite and nanocomposite samples was performed in two steps: first, the dispersion of nanostructured fillers into epoxy resin, and second curing of the mixtures. To execute the first step, the intended amount of each type of nanostructured filler for fabricating the composite samples (according to Table 1) was measured and added to the epoxy resin. Then, nanostructured fillers dispersion in the epoxy resin was conducted by an ultrasonic bath at 40˚C for 1 h. The prepared mixture was further dispersed using the mechanical stirrer at 40°C with a speed of 400 rpm for 1 h. Once again, the mixture was placed into an ultrasonic bath for 1 h. In order to eliminate air bubbles from the epoxy resin, the degassing process was performed by the vacuum desiccator for 30 min before adding the hardener. Subsequently, the hardener was added to the epoxy resin and completely mixed for 2 min. The obtained mixture was degassed one more time in a vacuum desiccator for 5min. After that, the resulting mixture was molded into an open lubricated silicone mold (For easy separation of samples), and the curing process was carried out at room temperature (30˚C) for 24 h. Finally, the dog-bone tensile test samples and wear test samples were made according to ASTM D638 and ASTM G99 respectively.

### 2.3 Experimental tests

The mechanical properties of pure epoxy and other nanocomposites investigated in this study were determined through the stress-strain curves obtained from the Instron hydraulic universal testing machine (model 8802) with a crosshead speed of 2 mm/min. The tensile tests were performed for at least 3 samples of each material at room temperature.

The friction and wear experiments were performed by using a pin-on-disc tribometer and conducted under dry contact conditions at room temperature according to ASTM G99. The tribometer was accurately calibrated for wear measurements. The 100 cr6 steel pin with a hardness of 58 HRC and spherical head with a diameter of 10 mm and length of 55 mm was employed as counterface. Prior to testing and measuring the weights of the samples, they were first cleaned and dried. For this purpose, acetone was used as a non-chlorinated, non-film-forming cleaning agent and solvent. Then, the pin was held by the pin holder and composite disc samples were screwed to the machine. Vertical loads with different values (20, 60 and 100 N) were applied to the specimens at each stage. The tests were carried out at a constant sliding velocity of 0.1 m/s and sliding distance of 1000 m and for wear track radius of 15, 20, and 25 mm in dry condition and room temperature. Each experiment was repeated 3 times to ensure the repeatability of the results and obtain the average values. The initial and final weights of the samples were measured using a balance with a precision of 0.0001 g (0.1 mg) to determine the weight loss. The friction force was measured using the wear test machine and then, the friction coefficient was obtained by dividing the friction force by the applied normal load.

## 3. Results and discussions

### 3.1 Tensile properties at room temperature

**Fig 1:**
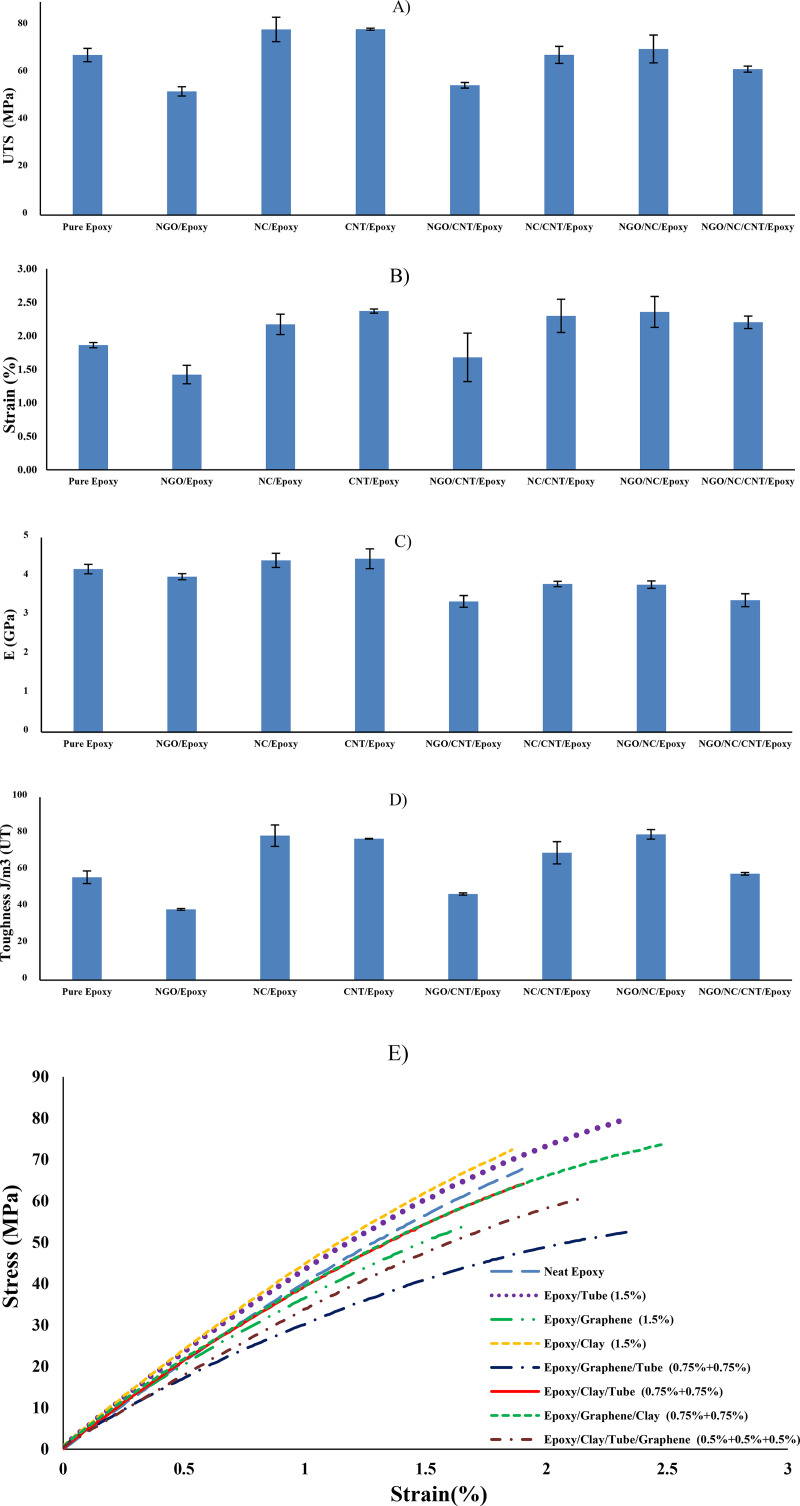
Pure epoxy and polymer matrix nanocomposites A) Ultimate Tensile Strength, B) Strain, C) Young modulus, D) Toughness, and E) Tensile stress-strain curve.

One of the main goals of the present study was to investigate the changes in mechanical properties due to the addition of NC, NGO and CNT with different weight percentages (Table 1) compared to the pure epoxy sample. The mean value along with the standard deviation, which describes how the test data are scattered around their mean, is shown in Fig 1. Some of the most important mechanical properties such as Young’s modulus, ultimate tensile strength, strain at the fracture point, and fracture toughness have been derived from the tensile test listed in Table 2.

**Table 2:** Mechanical properties of pure epoxy and nanocomposites.

Samples	Young modulus *E_c_* (GPa)	Tensile strength *σ_c_* (MPa)	Strain ε_b_(%)	Toughness (J/*m*^3^)
Pure Epoxy	4.19^±0.23^	67.57^±5.53^	1.86^±0.08^	56.24^±6.82^
NGO/Epoxy	3.99^±0.16^	52.21^±3.84^	1.42^±0.27^	38.67^±0.92^
NC/Epoxy	4.41^±0.35^	78.38^±10.09^	2.17^±0.29^	78.98^±11.43^
CNT/Epoxy	4.45^±0.49^	78.48^±0.86^	2.37^±0.06^	77.30^±0.35^
NGO/CNT/Epoxy	3.36^±0.29^	54.82^±2.33^	1.68^±0.71^	47.06^±1.18^
NC/CNT/Epoxy	3.81^±0.14^	67.64^±7.04^	2.30^±0.49^	69.63^±11.90^
NGO/NC/Epoxy	3.79^±0.18^	70.14^±11.50^	2.36^±0.45^	79.72^±5.08^
NGO/NC/CNT/Epoxy	3.39^±0.33^	61.61^±2.51^	2.20^±0.18^	58.15^±1.37^

Generally, it can be said that NC/epoxy and CNT/epoxy samples present better results compared to other composites while NGO/epoxy and NGO/CNT/Epoxy ones show a remarkable reduction in their most mechanical properties.

As shown in Fig 1, the highest tensile strength is related to CNT/epoxy sample, increased by about 16% compared to pure epoxy while there is a significant decrease of about 22.7% in the ultimate tensile strength of the NGO/epoxy sample compared to pure epoxy that can prove the results of Cicero [35] about NGO. It seems that CNT not only increased young modules but also increased the ductile mode of epoxy and consequently its toughness. Although CNT/epoxy sample shows good improvement in toughness, higher strain and Young’s modulus helps NGO/NC/epoxy and NC/epoxy samples respectively to obtain the highest toughness values compared to other composites. On the other hand, the results reveal that NGO/Epoxy sample has the lowest tensile strength and strain leading to a considerable decline in toughness value among other samples. These low strengths can be attributed to the inevitable aggregation and trend of graphene to bend/buckle. This degradation leads to a weak interfacial adhesion between matrix and filler which limits the improvement of mechanical properties [36].

Thus, only adding NGO) to epoxy exhibits more brittle behavior and consequently decreases all mechanical properties compared to pure epoxy, while adding NGO along with NC to epoxy has a profound impact on improving composite mechanical properties. This can be due to a higher degree of intercalation/exfoliation and better dispersion of NC particles that can modify the undesirable effects of NGO aggregation. Additionally, adhesively bonded joint of NC prevented interfacial failure and effectively increased its tensile strength. Moreover, some studies indicated that the addition of NC to composite can retard crack propagation due to bifurcation around the NCs and consequently enhance its fracture toughness and strength [37].

In return, it seems that the presence of CNT in epoxy structure enhances output results and there is no need to add additional fillers.

### 3.2 Wear behavior at room temperature

#### 3.2.1 Weight loss

**Fig 2:**
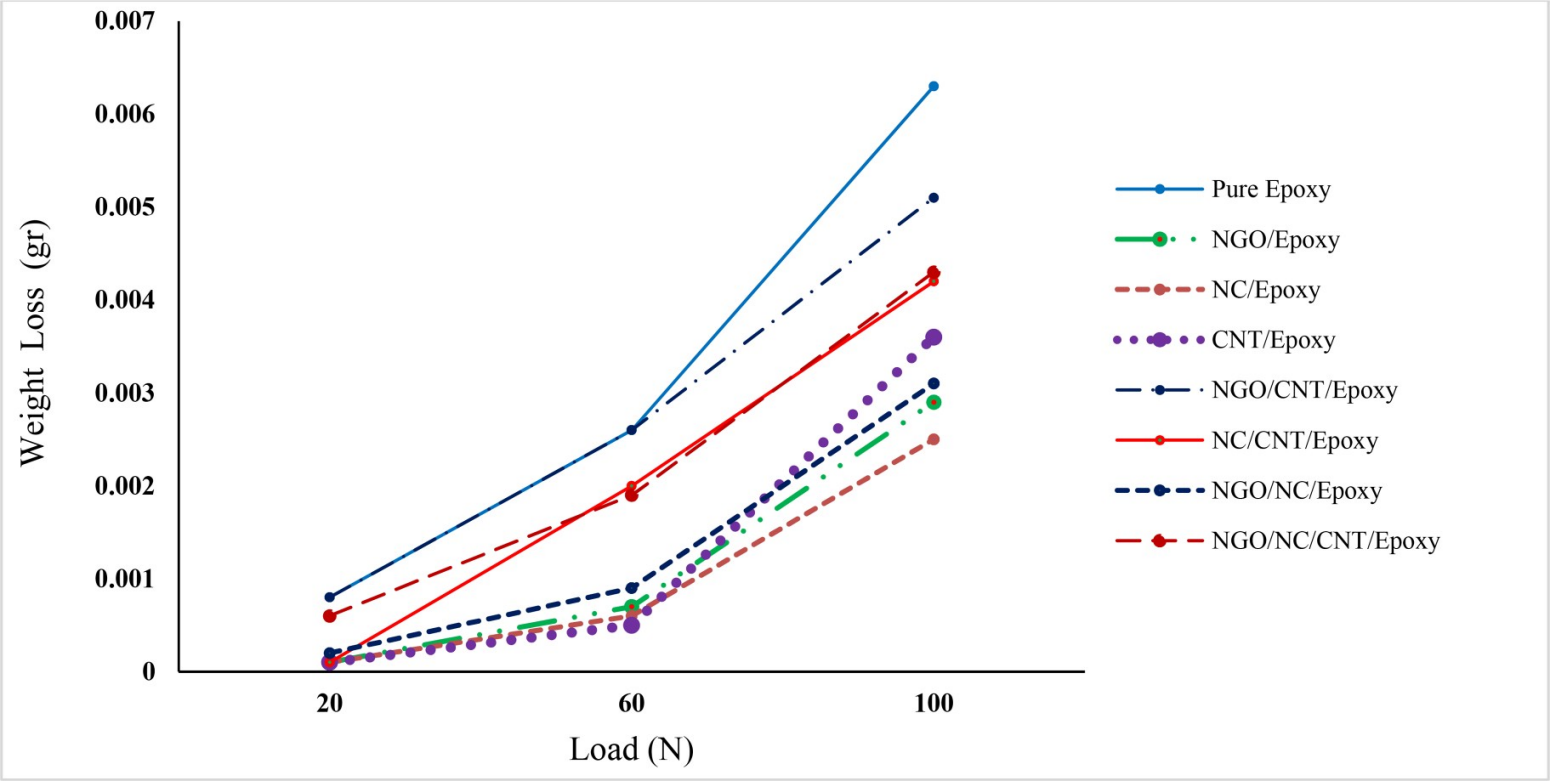
Weight loss of the studied materials in different applied loads.

The weight loss of the various nanocomposites was studied in this research. The results showed that at three different loads of 20, 60, and 100 N, most nanocomposites had significantly lower weight loss than that of pure epoxy (Fig 2). The nanocomposites containing only single-type fillers and also NGO/NC/epoxy at a load of 20N have lower weight loss with the almost same value. Although increasing load raised weight loss in all samples, NGO/epoxy, NC/epoxy, CNT/epoxy, and NGO/NC/epoxy presented again better results than other nanocomposites. Comparing these samples indicated that at the load of 60N, CNT/epoxy revealed the lowest weight loss value while at 100N, NC/epoxy showed the best value. Among nanocomposites with two and three types of fillers, NGO/NC/epoxy showed a remarkable increase in weight loss, proving this idea that adding NGO along with NC to epoxy presented acceptable outputs. In return, there was no considerable improvement when both NGO and CNT had been added to epoxy.

The weight loss of NGO/epoxy compared to pure epoxy is about 12.5, 35, and 46% for loads of 20, 60, and 100N, respectively. The wear rate of NC/epoxy for the aforementioned loads compared to pure epoxy is 12.5, 30, and ~40%, and these values for NC/NGO/epoxy are 25, 45, and 49%, compared to pure epoxy for the same conditions, respectively. This phenomenon might be due to the effect of the formation of the transfer film on the counterface during the wear process of the nanocomposites, which protects the soft polymeric material against the hard metallic material. Generally, the NC/epoxy nanocomposite samples show minimum weight loss, compared to other nanocomposites. The mechanical properties of the nanocomposites might be increased by the homogeneous distribution of the studied nanoparticles in the epoxy matrix. The increase in the interface local temperature will result in the adhesion of the materials to the steel pin and increasing the wear rate. The increase in weight loss by increasing load may also be attributed to the increase in the interface local temperature.

#### 3.2.2 Specific wear rate

The weight loss and specific wear rate (*W_r_*) of pure epoxy and nanocomposite samples are calculated with different applied normal loads under dry conditions. It is observed that weight loss increases as applied normal loads increase. The specific wear rate of samples is calculated as [38]:

$$W_{r}=\frac{\Delta m}{F_{n}\times \rho \times S}(\frac{{mm}^{3}}{N.m})$$ (1)

Where *Δm* is weight loss and *ρ* is the density of the samples and *F*_*n*_ and *S* are the applied normal load and sliding distance, respectively.

The measured wear rate as a function of the applied load for pure epoxy and other nanocomposites in three different loads (20, 60, and 100N) with a constant velocity of 0.1m/s and a sliding distance of 1000m are shown in Table 3. The studied nanocomposites have less weight loss than pure epoxy.

**Table 3:** Variation of wear rate (×10^−5^ mm^3^/N.m) at different loads.

Samples	20N	60N	100N
Pure Epoxy	3.20	3.46	5.04
NGO/Epoxy	0.40	0.93	2.32
NC/Epoxy	0.40	0.80	2.00
CNT/Epoxy	0.40	0.60	2.88
NGO/CNT/Epoxy	3.20	3.46	4.08
NC/CNT/Epoxy	0.40	2.66	3.36
NGO/NC/Epoxy	0.80	1.20	2.48
NGO/NC/CNT/Epoxy	2.40	2.53	3.44

At the load of 20N, CNT/epoxy, NC/epoxy, NGO/epoxy, and NC/CNT/epoxy samples had the same wear rate. Among nanocomposite samples, the nanocomposite containing 1.5 wt% CNT at the load of 60N, and the sample with 1.5 wt% NC at the load of 100N had the lowest wear rate, compared to other samples. Among all nanocomposites, the highest wear rates at the studied loads were recorded by NGO/CNT/epoxy sample.

#### 3.2.3 Coefficient of friction

**Fig 3:**
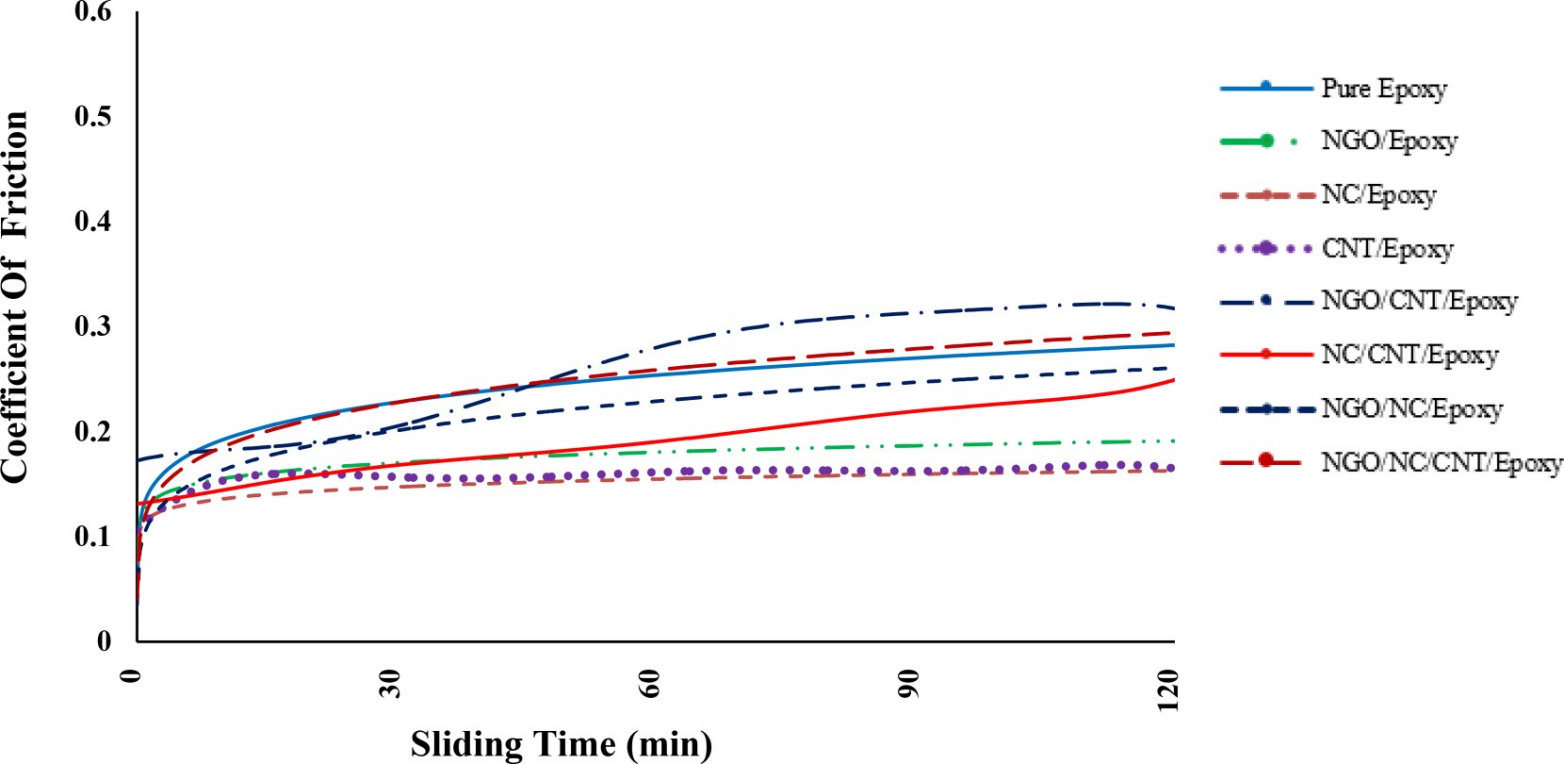
Variation of the coefficient of friction (μ) with sliding time.

Fig 3 shows the relation between the sliding time and coefficient of friction. Generally, there is an increase in the coefficient of friction as sliding time increases. The initial increase in the coefficient of friction can be due to not forming a transfer film under dry conditions. After that, the rate of increase in the coefficient of friction decreases, and values remain almost constant in some nanocomposites. This is due to the formation of the transfer film on the steel pin of the wear testing machine during each experiment. The transfer film is placed between the surface of the samples and the abrasive, which reduces wear and coefficient of friction. The friction coefficient is calculated using the following equation [39]:

$$\mu =\frac{F_{p}d_{p}}{F_{N}d_{N}}$$ (2)

where, *μ* is the friction coefficient, *F*_*p*_ and *F*_*N*_ are the rate of angular friction force and applied normal load, respectively. *d*_*p*_ and *d*_*N*_ are also the distance between the center to the pin and distance the center to the normal force, respectively. As shown, the samples consisting of a combination of two or three nanostructured fillers have a higher coefficient of friction while epoxy with just single-type filler presents less coefficient of friction value as sliding time exceeded 30 min compared to pure epoxy. As the experiment time increases, the coefficient of friction may increase due to the agglomeration of the nanostructured fillers and their accumulation in the epoxy matrix.

### 3.3 Morphology

Fracture and wear surfaces of the samples are examined to determine the failure and wear mechanisms using images obtained from FESEM using sputter-coating of the samples with a thin layer of gold.

#### 3.3.1 Fractured surfaces

**Fig 4:**
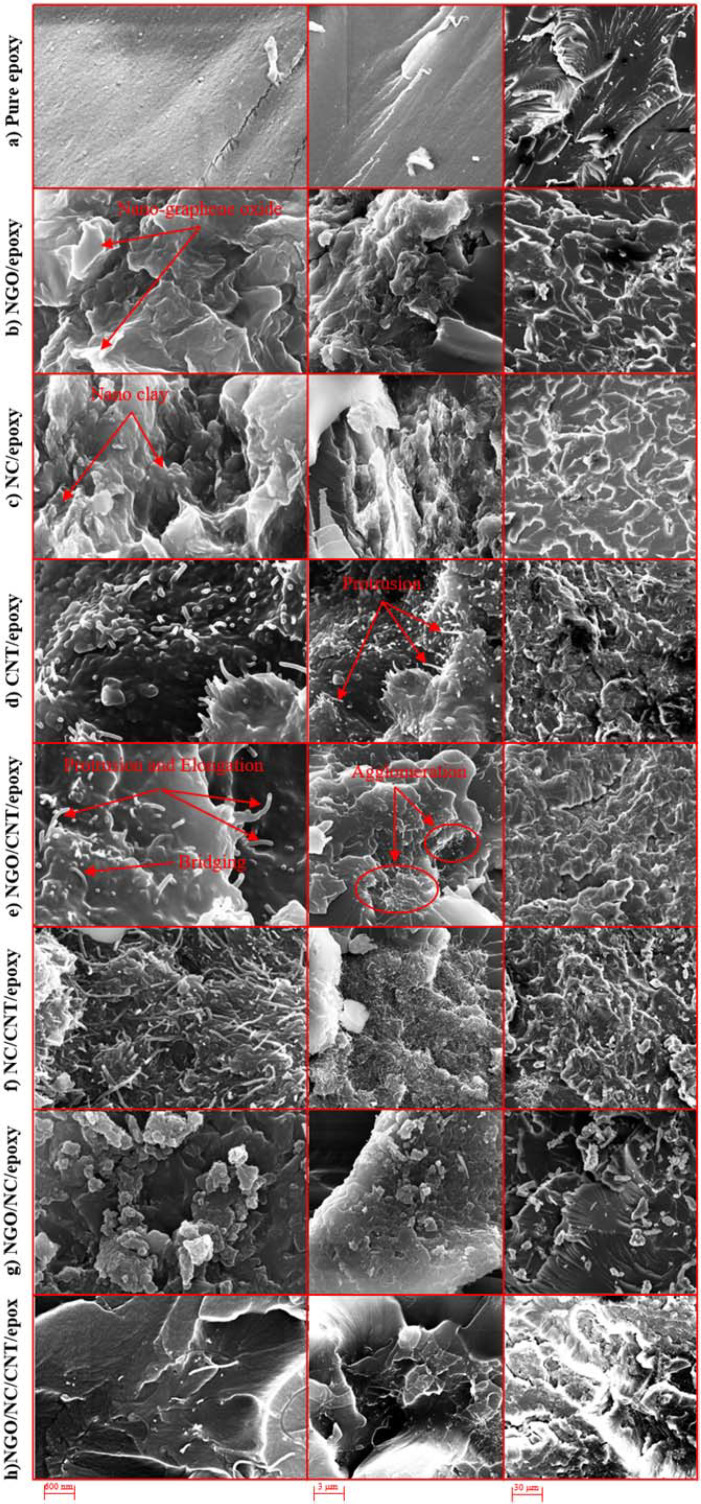
FESEM images of the fractured cross-sections of a) pure epoxy, b) NGO/epoxy, c) NC/epoxy, d) CNT/epoxy, e) NGO/CNT/epoxy, f) NC/CNT/epoxy, g) NGO/NC/epoxy, and h) NGO/NC/CNT/epoxy.

FESEM images of the fractured surfaces of pure epoxy and other nanocomposites at three different magnifications for each sample are shown in Fig 4. The fractured surface in pure epoxy is completely smooth (Fig 4A), the smooth movement of the layers in the epoxy sample surface indicates a ductile fracture of the sample.

For NGO/epoxy, the fracture surface is rough (Fig 4B). The image shows that the weak interface between NGO and epoxy prevents the transfer of load properly which leads to a reduction in its mechanical properties, Therefore, the NGO/epoxy nanocomposite exhibits mostly weaker properties than even pure epoxy. The addition of NGO to epoxy has increased the cracks at the fracture surface. Furthermore, the high content of the NGO in the nanocomposite may decrease the distances between the fillers, and agglomeration of the fillers may occur. The improper distribution of the nanostructured fillers throughout the matrix can finally reduce the strength of the nanocomposite. Also, small holes that are visible on the fracture surface indicate that NGO has the ability to nucleation of the holes. On the other hand, the brittle nature of NGO reduces the elongation at the breaking point. FESEM images show that NGO is generally non-uniform and randomly distributed in the matrix.

The mechanism of crack formation in a sample containing NC is completely different from that of pure epoxy due to the cohesion of the particles (Fig 4C), Therefore, adding NC to the epoxy improves the mechanical properties of pure epoxy. In this sample, a good bond between the nanoparticles and the matrix leads to crack growth resistance. Thus, the failure energy is increased and the failure resistance is improved; Consequently, compared to other samples, the NC/epoxy sample after the CNT/epoxy sample possesses the best fracture toughness.

For CNT/epoxy sample, the mode of the CNTs protrusion after loading indicates a strong bond between the epoxy and the CNTs. As shown in Fig 4D, the uniform distribution of CNTs filaments in the epoxy help to improve the tensile strength of the sample by their stretching and bending and prevent cracking in epoxy. Nevertheless, in all CNTs-containing samples, some CNTs have been pulled out of the fracture surface indicating the bridging mechanism of CNTs due to poor bonding between the CNTs and the epoxy. The fractured surface of CNTs-containing epoxy is rougher than the surface of pure epoxy. Small and scattered cracks are observed in the fractured surfaces of the CNTs-containing nanocomposite.

Regarding the NGO/CNT/epoxy sample, unlike CNT/epoxy sample, no uniform distribution of CNTs can be observed (Fig 4E). It seems that NGO influences the distribution of CNTs and results in agglomeration of the nanostructured fillers. These agglomerations create a stress concentration zone and reduce the tensile strength of the nanocomposite as shown in Fig 4E. The protrusion and bridging of the nanoparticles are also clearly visible. The tensile test results for this nanocomposite show 18.9% and 16.3% reduction in tensile strength and toughness compared to pure epoxy, respectively.

In the NC/CNT/epoxy sample, although CNTs are much more uniformly distributed in the epoxy sample than the NGO/CNT/epoxy without agglomeration around the NC (Fig 4F), the existence of NC cannot help to improve the cohesion of CNT in matrix. This can be due to the different geometry of both fillers. Thus, CNT epoxy performs better than NGO/CNT/epoxy and NC/CNT/epoxy samples.

On the other hand, the addition of NC to NGO increases the strength of the NGO/NC/epoxy sample and also may reduce the slip of graphene layers in the epoxy matrix (Fig 4G). NC increases the surface interaction between the matrix and the nanoparticles by being placed between the graphene surfaces. These factors and the uniform distribution of the nanostructured fillers in the epoxy matrix affect the mechanical properties of this sample.

The proper dispersion of the nanostructured fillers is seen in the NGO/NC/CNT/epoxy sample images in Fig 4H. The protrusion of the CNTs indicates that the load is tolerated by these nanostructured fillers. A small number of the holes can be seen on the fracture surface of this sample which indicates the uniform distribution of the nanostructured fillers.

#### 3.3.2 Wear surfaces

**Fig 5:**
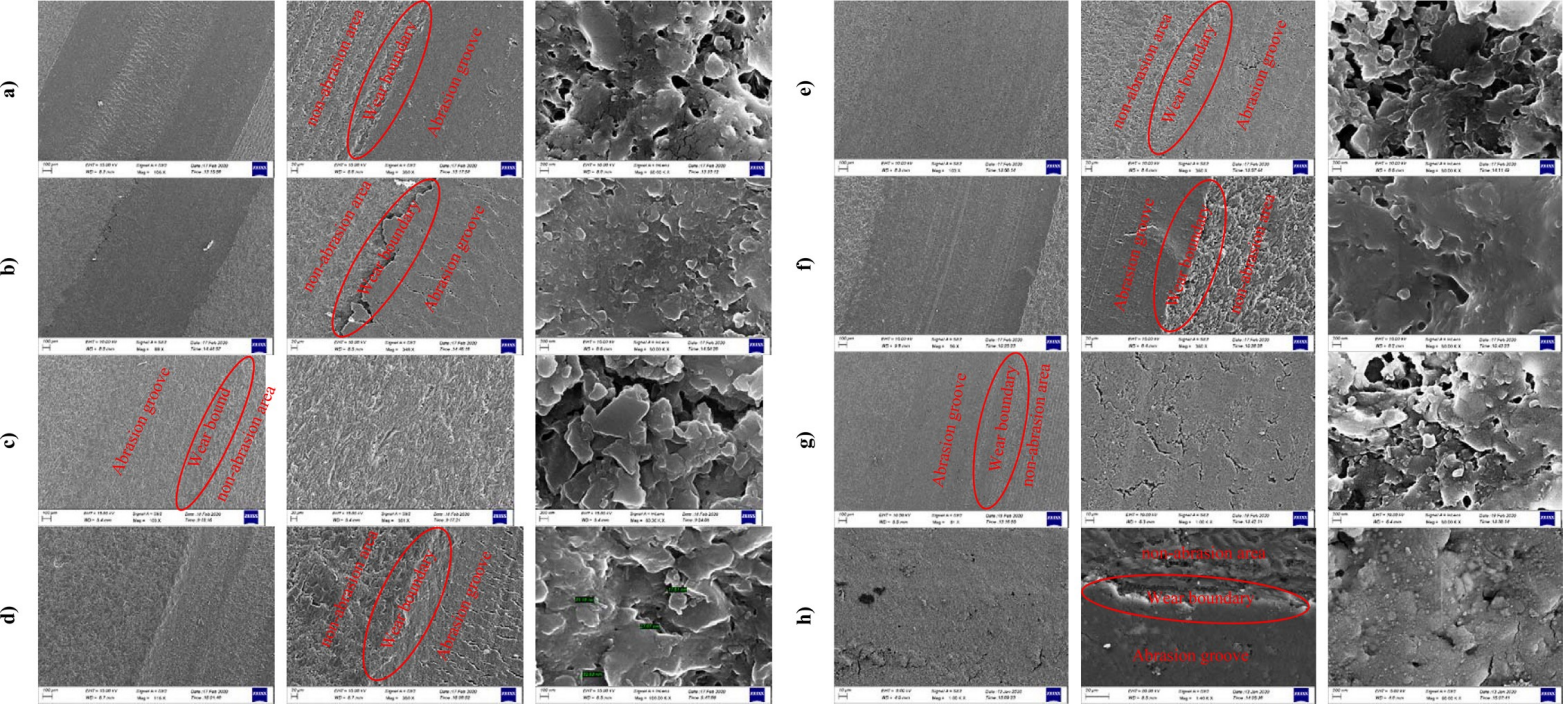
FESEM images of the wear surfaces (cross-sections) a) pure epoxy, b) NGO/epoxy, c) NC/epoxy, d) CNT/epoxy, e) NGO/CNT/epoxy, f) NC/CNT/epoxy, g) NGO/NC/epoxy, h) NGO/NC/CNT/epoxy.

Fig 5A shows the wear surface of the pure epoxy. Micrometer-sized cracks can be seen on the wear surface of the epoxy sample. Abrasion grooves in pure epoxy samples are wider and deeper than in other samples. Plastic deformation and resin peeling can be seen in the images. The wear mechanism in this sample is adhesive wear.

Debris particles produced during the abrasion process for the NGO/epoxy sample are smaller than other samples (Fig 5B). The main wear mechanism in this sample is typical fatigue wear. The repeated loading and unloading of contact stress eventually leads to fatigue fracture. Its characteristic feature is accumulation of irreversible changes, which give more generation and growth of cracks. The same results have been observed in [40,41].

The abrasion groove in the NC/epoxy sample is difficult to be detected because the wear depth in this sample is very small (Fig 5C).

By adding CNTs to the resin, wear on the abrasive surfaces is significantly reduced and the abrasion resistance of pure epoxy is significantly increased (Fig 5D). This can be due to the reduction of the coefficient of friction because of the lubricating properties of CNTs in the epoxy resin [42,43]. Adding the CNTs to epoxy increases the hardness of the nanocomposite, which ultimately leads to an enhancement in the wear properties of the nanocomposite. The protrusion and distribution of CNTs and abrasion residues are the most important wear mechanism in samples containing CNTs. Comparison between CNT/epoxy and NC/epoxy indicates that the CNT/epoxy composite has more significant cohesion. The addition of CNTs to epoxy resin significantly improves its wear behavior (Fig 3).

The composition of the NGO and CNTs increase the coefficient of friction so that the coefficient of friction of NGO/CNT/epoxy is even higher than that of pure epoxy. Increasing the coefficient of friction causes more weight loss of the sample due to wear peeling. Particles separated by wear are a factor in increasing the wear rate [44,45]. Due to the porosities that are seen in Fig 5E, there is a strong probability that abrasive particles separate the material from the wear surface as some research has already mentioned [46]. These images show the abrasive wear mechanism in the sample. This composite has the largest wear groove after pure epoxy.

The NC/CNT/epoxy sample has a higher coefficient of friction than the NC/epoxy and CNT/epoxy samples (Fig 3). The boundary between the abrasion groove area and the non-abrasion area is seen in FESEM images (Fig 5F). Micro-cracks and cavities resulting from the contact between the abrasive body and the sample can be seen on the sample surface.

Holes and micro-cracks are more visible in the NGO/NC/epoxy sample (Fig 5G). Parallel scratches are seen in the direction of abrasion. This is a common wear pattern observed in other abrasion studies [47,48]. Particles and debris separated by wear cause parallel grooves and remnants of wear can be seen in the corresponding FESEM image. It seems that although adding NC to NGO/epoxy helps to retard crack propagation due to branching around the NCs and enhance the fracture toughness of the composite, the existence of these two fillers with different properties in the composite can increase delamination and porosity on the surface during wear test. This may increase the friction coefficient significantly [49].

The worn surface of the NGO/NC/CNT/epoxy sample has signs of adhesion and abrasion (Fig 5H). The morphology of the surfaces shows signs of peeling due to the agglomeration of the nanostructured fillers in the matrix. This damage is caused by the agglomeration of the nanostructured fillers and residues of abrasive material. The boundary between the worn path and the non-abrasive surface is considerable due to the removal of a large amount of the resin.

## 4. Conclusion

Due to the importance of studying the abrasion damages on various engineering structures, in this research, the effect of nanostructured fillers with different weight percents on the mechanical and wear resistance properties of polymer matrix nanocomposites was investigated. For this purpose, NGO, CNTs, and NC with different weight percentages were used. Young’s modulus, ultimate tensile strength, strain at the breakpoint, and fracture toughness, coefficient of friction, weight loss, and specific wear rate were studied. It was found that NGO/epoxy had the lowest ultimate tensile strength, strain at the breakpoint, and toughness while NGO/epoxy nanocomposite showed more brittle fracture behavior than even the pure epoxy. The addition of CNTs to the epoxy resulted in the highest ultimate strength and the lowest tensile strength. It was observed that the highest amount of toughness was related to the NGO/NC/epoxy sample and the lowest amount of toughness was recorded for NGO/epoxy. This means that the synergy of NC and NGO had a significant effect on increasing toughness.

NC/epoxy nanocomposite demonstrated minimal weight loss compared to other nanocomposite samples. Among all nanocomposites, the highest wear rate was recorded for the NGO/CNT/epoxy sample. The NGO/CNT/epoxy sample showed the worst performance in terms of abrasion due to the agglomeration of the nanostructured fillers.
